# A Stimulus-Resolved Framework for Investigating Putative Neurotrophic Secretome–Metabolome Coupling in Aging Skeletal Muscle: CNTF and CLCF1 as Non-Equivalent Nodes

**DOI:** 10.3390/metabo16080562

**Published:** 2026-08-09

**Authors:** Fei Tong, Yirui Chen, Hongxin Gui, Aowei Li, Hongyu Li, Yusen Pei, Zimu Wu, Mengyang Wang

**Affiliations:** 1 College of Physical Education and Health, Tianjin University of Traditional Chinese Medicine, Tianjin 301617, China; 17695989338@163.com; 2 School of Public Health and Health Sciences, Tianjin University of Traditional Chinese Medicine, Tianjin 301617, China; ciandata@163.com (Y.C.); ghostcixi@163.com (H.G.); aowei.li@tjutcm.edu.cn (A.L.); hongyu.li@tjutcm.edu.cn (H.L.); yusen.pei@tjutcm.edu.cn (Y.P.); 3 School of Public Health and Preventive Medicine, Monash University, 553 St Kilda Road, Melbourne, VIC 3004, Australia

**Keywords:** skeletal muscle aging, metabolomics, secretome, CNTF, CLCF1, CRLF1, metabolic flexibility, exercise

## Abstract

Resting myokine abundance cannot distinguish adaptive signaling from compensation, tissue injury, altered receptor availability, or non-muscle contribution. We conducted a targeted narrative review of PubMed/MEDLINE, Embase, Web of Science Core Collection, and Scopus through 30 June 2026 to examine ciliary neurotrophic factor (CNTF), cardiotrophin-like cytokine factor 1 (CLCF1), and muscle metabolism across basal, insulin-stimulated, exercise, recovery, and training states. CNTF has the better-established CNTFRα–LIFR–gp130 receptor model, and pharmacological studies link it to AMPK activation, glucose uptake, ceramide handling, and insulin responsiveness. CLCF1 depends partly on CRLF1-associated extracellular availability, whereas its receptor usage and tissue source in skeletal muscle remain incompletely resolved. One recent study provides important preclinical and exploratory human evidence for exercise-responsive CLCF1, but direct human studies pairing CNTF/CLCF1 kinetics with muscle metabolomics or isotope-resolved flux are lacking. We, therefore, present neurotrophic secretory flexibility as a testable, hypothesis-generating construct rather than a validated biological system or biomarker. Its minimum evaluation requires synchronized measurements of extracellular ligand, receptor-proximal signaling, and metabolic output within the same physiological challenge. Targeted and untargeted metabolomics, lipidomics, quality-controlled annotation, paired tissue and plasma sampling, and stable isotope tracing can determine whether putative coupling is reproducible, muscle-relevant, and altered by aging.

## 1. Introduction

Skeletal muscle is the largest insulin-responsive metabolic organ and a major site of substrate switching. It is also an endocrine, paracrine, and neuromuscular communication tissue: myokines and exerkines can signal to adipose tissue, liver, bone, vasculature, immune cells, brain, peripheral nerves, motor neurons, and muscle itself [1,2,3,4,5]. Aging challenges this dual metabolic–communication role through mitochondrial dysfunction, altered mitophagy, denervation–reinnervation, sarcopenia, frailty, and sometimes obesity-related lipid stress [6,7,8,9,10,11,12]. These conditions overlap clinically but should not be treated as biologically interchangeable.

**Figure 1:**
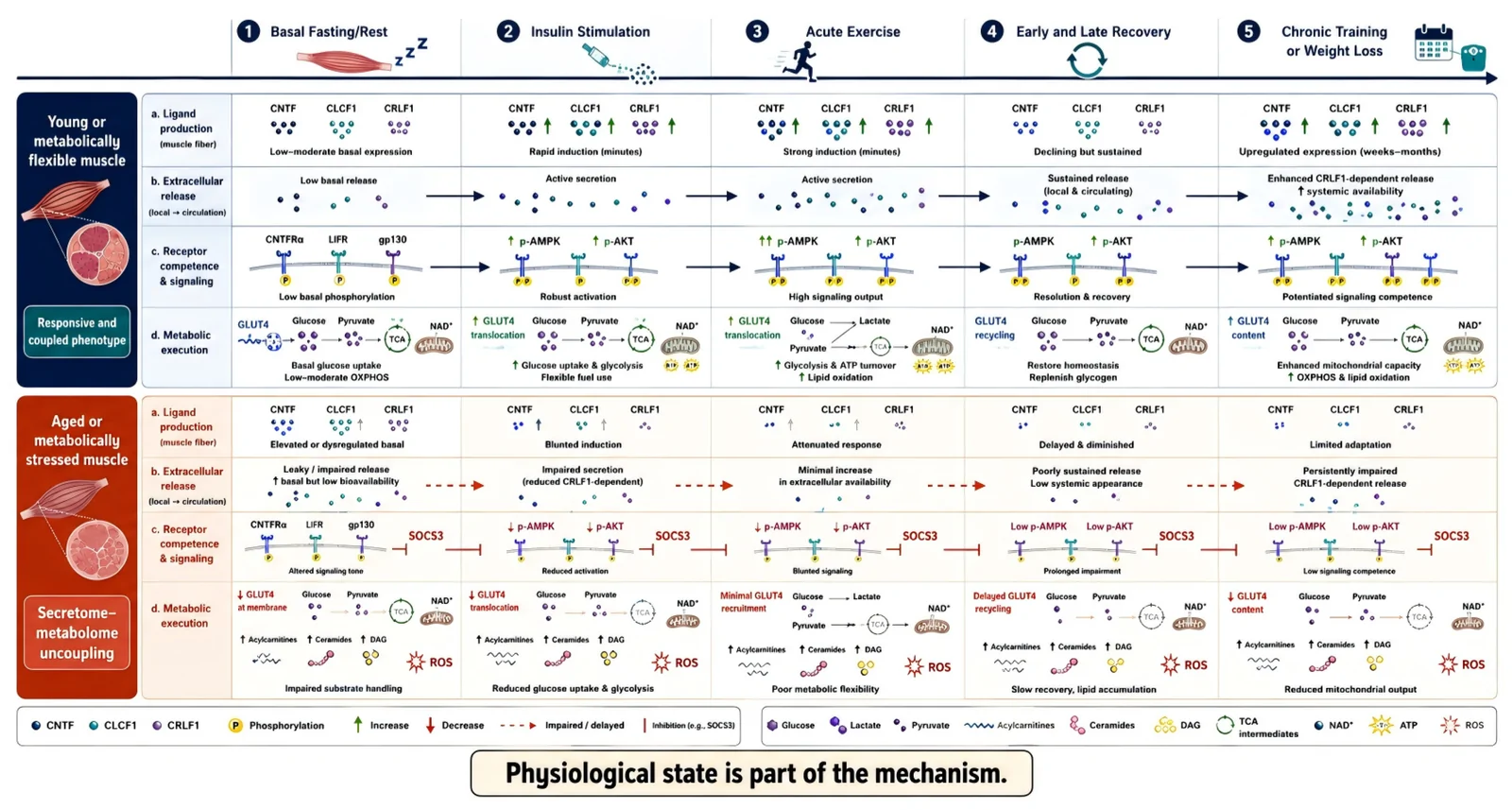
Conceptual stimulus-resolved model for investigating putative neurotrophic secretome–metabolome coupling in skeletal muscle aging. Basal fasting/rest, insulin stimulation, acute exercise, recovery, and training or weight loss may expose different relationships among ligand production, extracellular release, receptor-proximal signaling, and metabolic output. Solid arrows denote direct component-level mechanistic support in the experimental contexts described in the text; dashed or impaired arrows denote inference or hypothesis, not validated coupling. Abbreviations and symbols: ATP, adenosine triphosphate; CLCF1, cardiotrophin-like cytokine factor 1; CNTF, ciliary neurotrophic factor; CRLF1, cytokine receptor-like factor 1; CNTFRα, ciliary neurotrophic factor receptor alpha; DAG, diacylglycerol; GLUT4, glucose transporter type 4; LIFR, leukemia inhibitory factor receptor; NAD^+^, oxidized nicotinamide adenine dinucleotide; OXPHOS, oxidative phosphorylation; P, phosphorylation; ROS, reactive oxygen species; TCA, tricarboxylic acid cycle. Upward and downward arrows denote increases and decreases, dashed red arrows denote impaired or delayed responses, and blunt-ended lines denote inhibition.

A fasting cytokine concentration, a single muscle transcript, or a basal culture readout may represent adaptive signaling, compensation, tissue injury, receptor resistance, or non-muscle contribution. Insulin stimulation, acute contraction, recovery, training, and energy restriction impose different hormonal, mechanical, redox, calcium, and nutrient contexts. Physiological state is, therefore, part of the mechanism rather than a background variable (Figure 1).

The ciliary neurotrophic factor (CNTF) and cardiotrophin-like cytokine factor 1 (CLCF1) literature connects neurotrophic biology, interleukin-6-type cytokine signaling, and substrate metabolism. Pharmacological CNTF can activate AMP-activated protein kinase (AMPK), stimulate muscle glucose uptake, and limit ceramide-associated insulin resistance [13,14,15]. CLCF1 extracellular availability is influenced by cytokine receptor-like factor 1 (CRLF1), and recent work links exercise-responsive CLCF1 to muscle, bone, glucose tolerance, glycolytic flux, and mitochondrial activity in aged mice [16,17,18]. These ligands are not interchangeable: CNTF has a more established CNTFRα–leukemia inhibitory factor receptor (LIFR)–gp130 model, whereas CLCF1 receptor usage, complex formation, and tissue source remain context-dependent.

Here, “CNTF/CLCF1 network” denotes partial sharing of receptor components and possible functional convergence on metabolic signaling; it does not imply a demonstrated interaction between the ligands or a unified secretion pathway. We use neurotrophic secretory flexibility as a hypothesis-generating framework for testing synchronized changes in extracellular ligand, receptor-proximal signaling, and metabolic output. Direct human evidence connecting these layers is scarce, so all coupling terminology is explicitly putative unless simultaneous measurements have been made.

## 2. Literature Search and Evidence Classification

A targeted narrative search was last run in PubMed/MEDLINE, Embase, Web of Science Core Collection, and Scopus on 30 June 2026, with coverage from database inception. Terms addressed the CNTF/CLCF1 network, skeletal muscle and aging, physiological challenges, and metabolic or omics readouts; platform-specific representative queries are reported in Appendix A. Crossref was used only for bibliographic verification. Manual reference chasing of key CNTF, CLCF1, gp130, myokine, exercise-metabolomics, and sarcopenia articles supplemented database retrieval and contributed older canonical receptor and pharmacological studies as well as selected contextual records. The retained PubMed export contained 695 records. Exact final executed strings, equivalent record-level exports, and complete search-history logs were not retained for every database; consequently, cross-database retrieval totals, duplicate counts, and a retrospective PRISMA-style flow cannot be reconstructed reliably. The Appendix A strings are, therefore, representative reconstructions rather than a verbatim search audit trail. We describe the process as a targeted narrative search, not a systematic or fully reproducible review.

Primary human, animal, cell, and ex vivo studies were prioritized when they connected a network component or a relevant physiological challenge to metabolic outcomes. Reviews positioned adjacent myokine, sarcopenia, mitochondrial, exercise, and nutrition evidence [19,20,21,22,23,24,25,26,27,28,29]. Detailed synthesis was limited to peer-reviewed full articles in English or with sufficient accessible English-language text and methods. Conference abstracts, dissertations, preprints, inaccessible full texts, and non-English records without adequate English-language information were not used as core evidence. Screening and classification were not conducted as blinded, independent duplicate systematic-review procedures; uncertain relevance was discussed within the author team. No formal risk-of-bias assessment or protocol registration was performed. These constraints limit completeness and preclude quantitative evidence grading.

Evidence was classified in practice as follows: direct component-level evidence measured a named CNTF/CLCF1-network component and a metabolic outcome in the same experiment; partial mechanistic evidence tested one receptor, signaling, secretion, or metabolic step without the complete construct; contextual evidence defined relevant aging, exercise, or metabolomic biology without testing CNTF/CLCF1 and hypothesis-generating statements proposed an untested relationship. No study measured the complete secretome–receptor–metabolome construct; the term “direct component-level”, therefore, does not denote direct evidence for complete coupling.

The nine selected core records in Table 1 were chosen purposively to provide a compact boundary-setting inventory: they had to be primary studies and collectively represent (i) named CNTF or CLCF1 components measured with metabolic outcomes, (ii) receptor or signaling steps needed to interpret those outcomes, and (iii) tissue-context findings that constrain systemic extrapolation. The table is not an exhaustive enumeration of the CNTF/CLCF1 or adjacent metabolic literature. Its counts—four direct component-level, three partial mechanistic, and two contextual boundary studies—apply only to this selected inventory, not to all 83 cited records, the 695-record PubMed export, or the wider literature base. Hypotheses are not counted as studies.

## 3. Molecular Architecture and Receptor Uncertainty

### 3.1. CNTF and CLCF1 Are Related but Not Interchangeable

CNTF contributes to motor-neuron support, neuromuscular biology, and muscle strength [35,36,37,38]. Its metabolic evidence is mainly pharmacological: recombinant CNTF and related gp130 ligands can activate AMPK and increase muscle glucose uptake in obesity-linked models [13,14,39,40,41]. This does not establish adaptive endogenous secretion in aging muscle. CNTF lacks a classical signal peptide, so extracellular abundance may also reflect non-classical release, fiber damage, denervation, or metabolic compensation.

CLCF1 follows a different secretion logic. Its extracellular availability is influenced by CRLF1-containing complexes, so intracellular expression need not predict interstitial or circulating exposure [17,18]. The recent exercise study linked CLCF1 to glucose uptake, glycolytic flux, mitochondrial activity, performance, and bone outcomes in aged male mice [16]. However, muscle-specific CLCF1 manipulation did not fully explain circulating concentrations, and source attribution remains unresolved.

The exploratory human component of that study should be interpreted precisely. It reanalyzed vastus lateralis transcriptomic data from young (24–25 years) and older (78–84 years) adults sampled around acute resistance exercise and a 12-week progressive resistance-training program. It also examined six small and heterogeneous plasma cohorts spanning acute resistance, jiu-jitsu-specific high-intensity interval, aerobic treadmill, chronic resistance, resistance-band, and basal age-comparison settings. The human measurements were muscle transcript reanalysis and plasma CLCF1 enzyme-linked immunosorbent assay, not paired human muscle metabolomics or isotope tracing. Available sample size, sex, age, modality, timing, material, assay, and unreported fields are summarized cohort by cohort in Supplementary Table S1 [16].

### 3.2. Canonical and Alternative Receptor Usage

The canonical CNTF configuration uses CNTFRα for ligand recognition and LIFR–gp130 for signal transduction. Here, gp130 denotes the receptor protein encoded by the human gene *IL6ST*. Suppression of gp130 protein attenuates insulin-mediated signaling and glucose uptake in muscle cells, whereas human *IL6ST* gene expression correlates with obesity and insulin sensitivity [30,31]. Related cytokine studies support JAK–STAT, ERK, PI3K–AKT, AMPK-linked, and suppressor of cytokine signaling (SOCS) pathways, but shared receptors also create pleiotropy and feedback-dependent desensitization [32,42,43,44,45].

**Figure 2:**
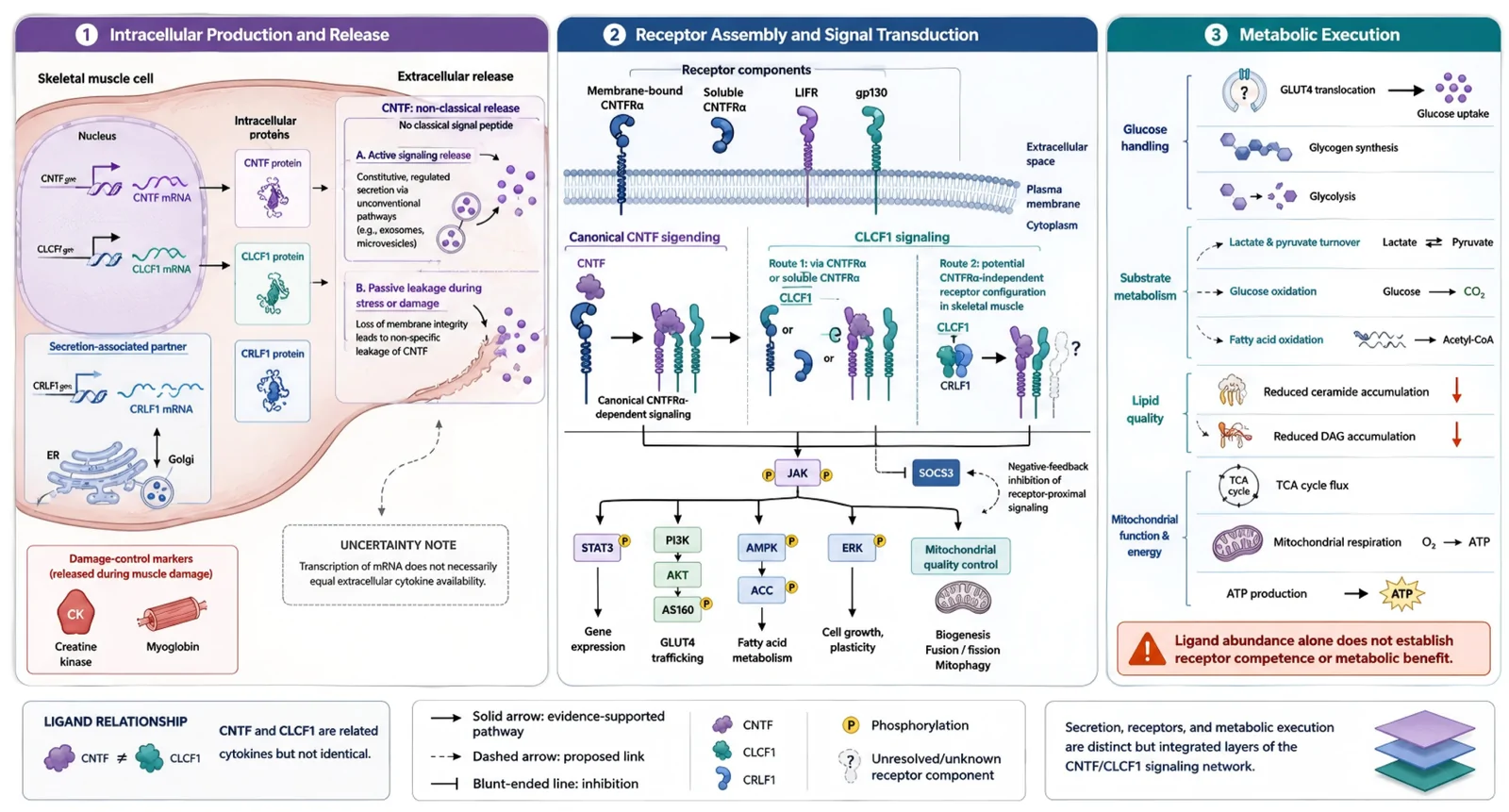
Molecular architecture used to distinguish established from unresolved receptor configurations. The CNTF–CNTFRα–LIFR–gp130 complex is the better-established model. CLCF1 extracellular availability can involve CRLF1 or soluble CNTFRα, whereas skeletal-muscle receptor use is cell-type- and complex-dependent; alternative LIFR–gp130 configurations remain proposed rather than confirmed. Downstream arrows indicate direct component-level evidence and do not establish complete secretome–metabolome coupling. Abbreviations and symbols: ACC, acetyl-CoA carboxylase; AKT, protein kinase B; AMPK, AMP-activated protein kinase; AS160, AKT substrate of 160 kDa; ATP, adenosine triphosphate; CK, creatine kinase; CLCF1, cardiotrophin-like cytokine factor 1; CNTF, ciliary neurotrophic factor; CNTFRα, ciliary neurotrophic factor receptor alpha; CO_2_, carbon dioxide; CRLF1, cytokine receptor-like factor 1; DAG, diacylglycerol; ER, endoplasmic reticulum; ERK, extracellular signal-regulated kinase; GLUT4, glucose transporter type 4; gp130, glycoprotein 130 protein encoded by *IL6ST*; JAK, Janus kinase; LIFR, leukemia inhibitory factor receptor; O_2_, oxygen; P, phosphorylation; PI3K, phosphoinositide 3-kinase; SOCS3, suppressor of cytokine signaling 3; STAT3, signal transducer and activator of transcription 3; TCA, tricarboxylic acid cycle. Solid arrows denote evidence-supported paths, dashed arrows denote proposed links, blunt-ended lines denote inhibition, and question marks denote unresolved receptor components. Red downward arrows denote decreases, and colors distinguish ligands and conceptual layers without encoding effect size.

CLCF1 can occur in CRLF1- or soluble CNTFRα-containing extracellular complexes. In skeletal muscle models, LIFR and gp130 appear important, whereas CNTFRα knockdown does not uniformly suppress CLCF1-induced STAT3, AKT, or ERK phosphorylation [16]. Thus, CNTFRα-dependent and alternative LIFR–gp130 configurations remain possible and may vary by cell type, extracellular complex, and dose. In this review, the network terminology, therefore, refers to shared components and functional convergence, not a demonstrated CNTF–CLCF1 interaction (Figure 2).

## 4. Operational Definition and Falsifiable Predictions

We define putative neurotrophic secretome–metabolome coupling as a reproducible, time-aligned association among three measured layers within the same experimental unit and physiological challenge: (1) extracellular CNTF or CLCF1 availability, with CRLF1 or ligand-complex status where relevant; (2) receptor-proximal response, such as phosphorylation of STAT3, AKT, AMPK, or ERK rather than receptor transcript abundance alone and (3) a quantitative metabolic output, such as glucose uptake or traced carbon fate, lipid-species remodeling, or mitochondrial respiration. Muscle transcript or intracellular protein alone cannot demonstrate secretion, and receptor abundance alone cannot demonstrate functional receptor competence.

At minimum, basal and at least two post-stimulus time points should be collected to estimate peak change, area under the response curve, time to peak, and recovery. Repeated visits are needed to quantify within-person variability. Active muscle release requires stronger source evidence than a plasma increase: interstitial sampling, arterial–venous balance, temporal precedence, or tissue-specific manipulation should be combined with creatine kinase, myoglobin, and other damage measures. These damage markers are supportive controls but cannot alone exclude leakage or non-muscle sources.

**Figure 3:**
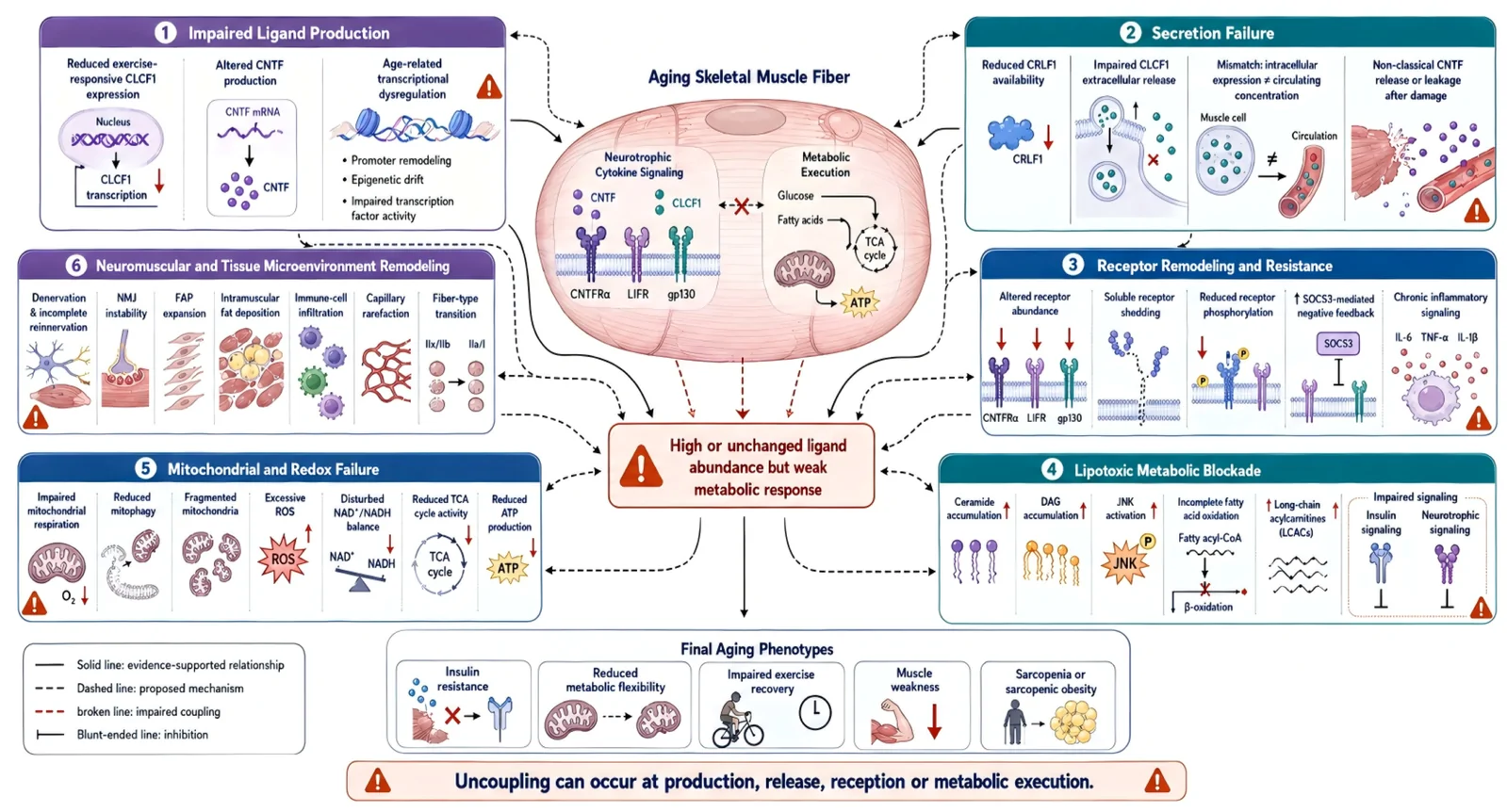
Hypothesis-generating breakpoints that could produce apparent or genuine uncoupling. Candidate mechanisms include impaired CRLF1-associated extracellular availability, context-dependent CLCF1 receptor usage, reduced LIFR–gp130 signaling, SOCS feedback, denervation, ceramide or diacylglycerol accumulation, incomplete fatty-acid oxidation, redox stress, and impaired mitochondrial quality control. None is established as a complete CNTF/CLCF1-to-metabolome pathway in aging human muscle. Abbreviations and symbols: ATP, adenosine triphosphate; CLCF1, cardiotrophin-like cytokine factor 1; CNTF, ciliary neurotrophic factor; CNTFRα, ciliary neurotrophic factor receptor alpha; CRLF1, cytokine receptor-like factor 1; DAG, diacylglycerol; FAP, fibro-adipogenic progenitor; gp130, glycoprotein 130; IL-1β, interleukin 1 beta; IL-6, interleukin 6; JNK, c-Jun N-terminal kinase; LCACs, long-chain acylcarnitines; LIFR, leukemia inhibitory factor receptor; NAD^+^/NADH, oxidized/reduced nicotinamide adenine dinucleotide; NMJ, neuromuscular junction; O_2_, oxygen; P, phosphorylation; ROS, reactive oxygen species; SOCS3, suppressor of cytokine signaling 3; TCA, tricarboxylic acid cycle; TNF-α, tumor necrosis factor alpha. Solid lines denote evidence-supported relationships, dashed lines proposed mechanisms, broken red lines impaired coupling, and blunt-ended lines inhibition. Upward and downward arrows denote increases and decreases, respectively; crosses denote blockades or failed coupling; and the not-equal sign denotes non-equivalence.

The construct is falsifiable. Repeated ligand changes without receptor-proximal or metabolic responses would refute integrated coupling in that context. Preserved metabolic adaptation after muscle-specific ligand/receptor disruption would argue that the proposed node is not necessary. A circulating response that lacks local release evidence, precedes no receptor activation, or is explained by tissue injury would refute a muscle-secretory interpretation. Figure 3 shows candidate breakpoints rather than established age-related mechanisms.

Table 2 converts the construct into minimum measurements, response metrics, and observations that would weaken or falsify it. The labels are experimental configurations, not clinical subtypes.

## 5. Direct Component-Level and Indirect Metabolomic Evidence

No study has produced a CNTF/CLCF1-specific metabolomic map of aging human muscle. Existing evidence is layered: network-specific studies provide targeted functional or biochemical endpoints; related receptor studies support individual signaling steps; aging-muscle and exercise metabolomics define contextual modules and several proposed metabolites remain prospective. Human exercise-mode metabolomics and adjacent lifestyle or secretome-intervention studies provide contextual exposure evidence [46,47,48,49,50]. Lipid burden and circadian/redox biology define additional measurement domains [51,52,53,54], while frailty, recovery, and exercise-responsive metabolites further establish state dependence [55,56,57,58,59,60]. Table 3 preserves these boundaries.

### 5.1. Glucose, Lipid, and Mitochondrial Modules

CNTF provides the clearest component-level bridge to glucose metabolism, but pharmacological glucose uptake is not equivalent to endogenous carbon reallocation [13,14]. Future studies should trace glucose into lactate/pyruvate, glycogen, TCA-cycle intermediates, and oxidation rather than infer fate from uptake alone. Lipid intermediates are equally important because ceramide, DAG, and incomplete beta-oxidation may blunt receptor signaling; CNTF can reduce ceramide-associated insulin resistance in an acute lipid challenge, whereas obesity impairs CNTF-stimulated glucose uptake [12,15,51,52,62,63,64]. AMPK can participate bidirectionally in muscle energy metabolism and myokine secretion [65]. Mitochondrial respiration, TCA intermediates, adenine nucleotides, NAD^+^ metabolites, and glutathione-related markers can distinguish acute signaling from durable remodeling, but these modules remain contextual rather than network-specific [6,7,8,10,53,54,66,67]. Studies of irisin and meteorin-like illustrate adjacent AMPK-linked myokine effects without establishing CNTF/CLCF1 specificity [68,69]. A recent synthesis linking mitochondrial redox dysfunction and ferroptosis across cardiometabolic disease further supports prespecified redox-sensitive handling while remaining outside the CNTF/CLCF1 evidence tier [70].

### 5.2. Analytical Platforms, Identification, and Quality Control

Untargeted liquid chromatography–mass spectrometry, gas chromatography–mass spectrometry, and nuclear magnetic resonance spectroscopy can discover state-dependent modules, whereas targeted assays are preferable for absolute or semi-quantitative measurement of acylcarnitines, ceramide/DAG species, amino-acid catabolites, nucleotides, and redox metabolites. Stable isotope tracers are required when pathway flux, rather than pool size, is the claim. Muscle and interstitial samples provide greater tissue proximity; plasma offers repeated sampling but integrates multiple organs.

Sample order should be randomized across age, sex, and state. Internal standards, extraction blanks, pooled quality-control samples, dilution series, and batch correction should be prespecified, with analytical coefficients of variation reported. Tissue data require normalization to wet weight, protein, DNA, or cell number as appropriate; plasma data require standardized anticoagulant, processing delay, hemolysis assessment, and storage history. Metabolite identities should be reported using explicit confidence levels: authentic-standard and retention-time confirmation for high-confidence claims, spectral-library matches for putative annotations, and feature-level labels when structures remain unknown. Pathway analysis should account for platform coverage, correlated features, multiple testing, and annotation uncertainty. Multi-omics integration should be performed only after modality-specific quality control and should report validation or stability, not only fitted latent factors.

Human aging and exercise studies show why these safeguards matter: frailty and sarcopenia can have distinct tissue signatures, exercise mode and intensity alter circulating profiles, and recovery windows reveal metabolites missed by a single post-exercise sample [11,46,47,55,56,57]. These studies justify the analytical roadmap but do not convert contextual metabolites into CNTF/CLCF1 biomarkers.

## 6. State-Resolved Evidence and Tiered Validation

Basal measurements remain useful for phenotyping but cannot identify adaptive reserve. Denervation can increase muscle CNTFRα expression, and fasting ligand levels may reflect injury, compensation, receptor resistance, or non-muscle contribution [38]. Insulin clamps or ex vivo insulin stimulation can reveal receptor and metabolic responsiveness, but oral glucose tolerance tests additionally incorporate incretin, perfusion, and substrate effects. Exercise must be prespecified by mode, intensity, duration, workload, damage, and recovery time because CLCF1 and metabolite responses are not uniform across resistance, interval, and endurance protocols [16,46,47].

Aging, obesity, and insulin resistance are related but non-equivalent contexts. The strongest CNTF metabolic studies model obesity or acute lipid exposure rather than aging [13,14,15]. They support candidate mechanisms, not age-specific causation. Conversely, the principal aging-focused CLCF1 study does not provide paired human muscle metabolomics. Analyses should, therefore, stratify age, adiposity, sex, insulin sensitivity, myosteatosis, and training status rather than use obesity as a surrogate for aging.

Physiological and pharmacological exposure also require separation. Recombinant ligand concentrations and exposure durations may exceed local endogenous pulses, engage shared gp130/LIFR pathways in multiple tissues, and provoke SOCS-mediated desensitization. Dose, duration, route, endogenous versus recombinant exposure, and time since prior stimulation should be reported explicitly. Adjacent FGF21 and broader myokine studies likewise show that hyperinsulinemia, training, exercise resistance, and detraining are separable exposure contexts [71,72,73,74]. Table 4 combines state selection, assay layers, and decisive comparisons into feasible study tiers; the most invasive tier is informative but not required for every question.

## 7. Biological Modifiers and Alternative Explanations

Chronological age should be separated from adiposity, insulin resistance, biological muscle age, and training history. Mitochondrial quality, myosteatosis, denervation, fiber type, sex hormones, diet, sleep, and circadian phase can each alter ligand concentrations or metabolic responses [11,23,26,51,53,54]. Processing-related dietary exposures and gut–liver–brain metabolic pathways may further modify the systemic metabolite background and should not be misattributed to muscle secretion [75]. Analyses should, therefore, be sex-stratified where possible and should report menopausal status, body composition, insulin sensitivity, habitual activity, nutrition, liver/metabolic phenotype, and last exercise bout.

Whole-muscle RNA or protein averages also mix fibers, stromal and endothelial cells, immune cells, motor-neuron-associated niches, and damaged tissue. Single-nucleus or spatial methods can localize production, but extracellular source still requires interstitial, arterial–venous, temporal, or tissue-specific evidence. A plasma association may reflect another organ, altered clearance, barrier function, or systemic inflammation rather than muscle secretion.

## 8. Conflicting Evidence and Limitations

The evidence base is both sparse and internally heterogeneous. CNTF can improve glucose handling in pharmacological obesity or lipid-challenge models, yet obesity also impairs CNTF-stimulated glucose uptake [13,14,15]. CLCF1 appears beneficial in aged male mouse muscle and bone, but brown-adipose studies report reduced thermogenesis and mitochondrial biogenesis [16,33,34]. These are not necessarily contradictions because tissue, dose, duration, receptor configuration, and physiological state differ. They do, however, preclude a uniformly beneficial systemic interpretation.

Direct component-level human evidence remains limited to exploratory CLCF1 observations and associative or intervention-related CNTF measurements. No study has simultaneously established muscle source, physiological ligand kinetics, receptor-proximal signaling, and metabolomic or isotope-resolved execution in aging humans. Muscle-specific CLCF1 manipulation does not fully account for circulating CLCF1; therefore, intracellular expression, extracellular release, local signaling, systemic concentration, and causal mediation of exercise adaptation must remain distinct claims.

Low-abundance cytokine measurement introduces additional uncertainty. Immunoassays may differ in antibody specificity, detection limits, matrix effects, free versus CRLF1- or receptor-complexed ligand recognition, freeze–thaw sensitivity, and inter-assay variability. Studies should report the assay platform, calibration range, lower limit of quantification, dilution linearity, spike recovery, duplicate precision, batch, and the proportion of values below quantification. Orthogonal validation is desirable when a key conclusion depends on a small concentration change.

Finally, this narrative review was not registered, did not use formal risk-of-bias assessment, and did not retain complete record-level logs for every database. Screening and evidence classification were not performed as independent duplicate systematic-review procedures. The 695-record PubMed export and 83 cited records provide an auditable partial inventory, but neither a PRISMA flow nor quantitative evidence certainty can be reconstructed. Selection bias and misclassification, therefore, remain possible, and the framework should be used for experimental design rather than effect estimation.

## 9. Multi-Omics Integration

**Figure 4:**
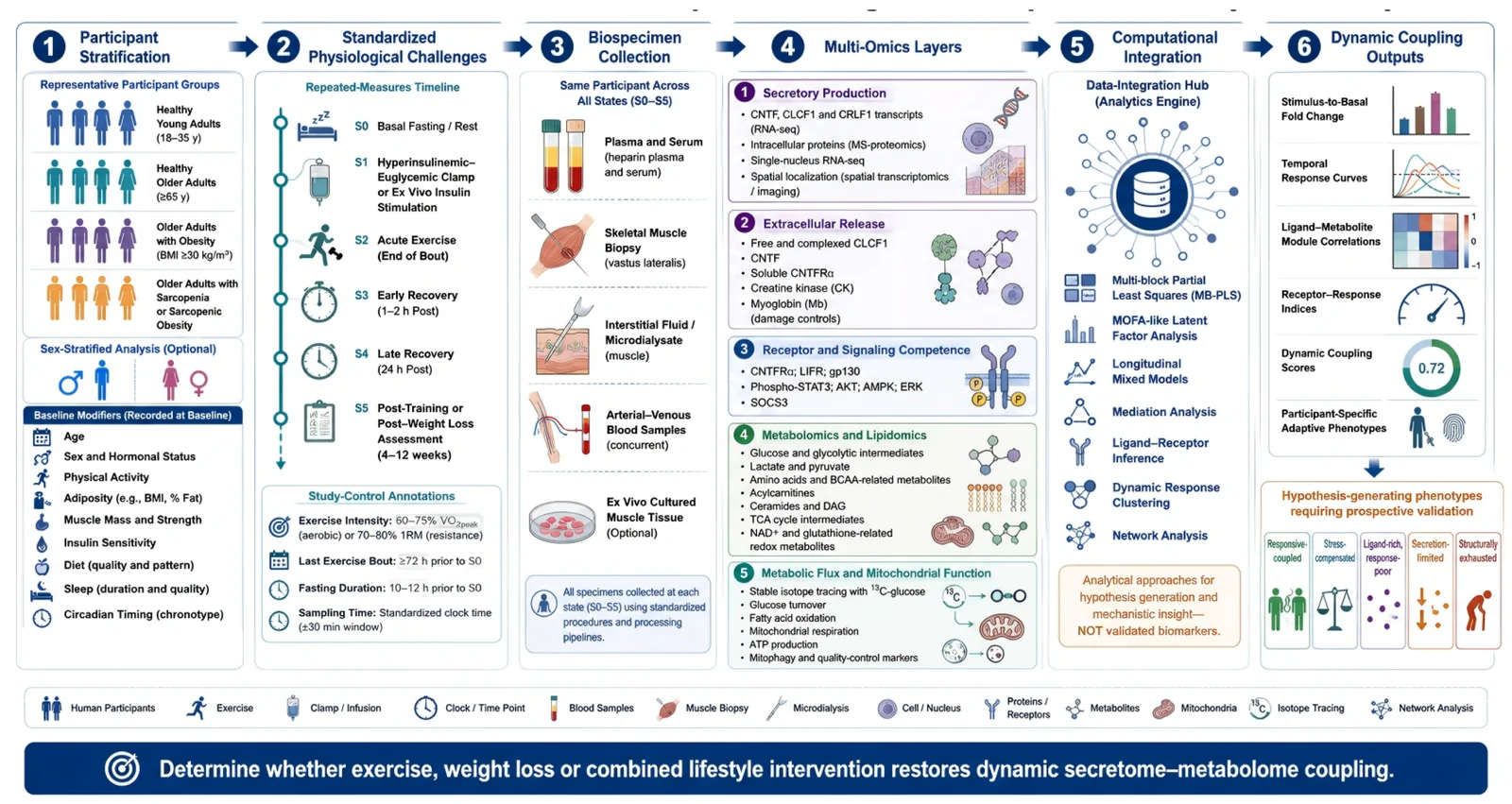
Comprehensive multi-state validation roadmap. The figure illustrates an aspirational program spanning fasting baseline, insulin stimulation, exercise, recovery, and training. Feasible studies may implement the tiers in Table 4 rather than every component. Paired plasma, muscle, interstitial fluid, phosphoproteomics, metabolomics, lipidomics, and stable isotope tracing can test source, temporal ordering, and metabolic flux. Abbreviations and symbols: ^13^C, carbon-13 isotope; AKT, protein kinase B; AMPK, AMP-activated protein kinase; BCAA, branched-chain amino acid; BMI, body mass index; CK, creatine kinase; CLCF1, cardiotrophin-like cytokine factor 1; CNTF, ciliary neurotrophic factor; CNTFRα, ciliary neurotrophic factor receptor alpha; CRLF1, cytokine receptor-like factor 1; DAG, diacylglycerol; ERK, extracellular signal-regulated kinase; gp130, glycoprotein 130; LIFR, leukemia inhibitory factor receptor; MB-PLS, multi-block partial least squares; Mb, myoglobin; MOFA, multi-omics factor analysis; MS, mass spectrometry; NAD^+^, oxidized nicotinamide adenine dinucleotide; RNA-seq, RNA sequencing; SOCS3, suppressor of cytokine signaling 3; STAT3, signal transducer and activator of transcription 3; TCA, tricarboxylic acid cycle; 1-RM, one-repetition maximum; VO_2peak_, peak oxygen uptake. S0–S5 denote baseline, insulin stimulation, acute exercise, early recovery, late recovery, and post-training or post-weight-loss states, respectively.

Figure 4 depicts a comprehensive research program rather than a mandatory design. A staged approach is more feasible: establish assay performance and repeatable ligand kinetics; add paired receptor phosphorylation and targeted metabolic outputs; then reserve biopsy-intensive multi-omics, arterial–venous sampling, microdialysis, and isotope tracing for focused causal questions. Multi-block partial least squares, factor models, mediation analysis, and dynamic clustering can integrate modalities, but training/validation separation, bootstrap stability, missing-data handling, and sensitivity to preprocessing should be reported. A latent factor is not evidence of causality unless its temporal and experimental predictions are independently tested.

## 10. Clinical and Translational Implications

No clinically validated CNTF/CLCF1 response index exists. Basal or stimulated CNTF, circulating CLCF1, CLCF1/CRLF1, soluble CNTFRα, and coupling coefficients are research variables only. Their assay validity, tissue origin, biological variability, test–retest reliability, clinically meaningful range, and external reproducibility remain unknown.

The framework may help structure trials of heterogeneous lifestyle responses, but it should not guide individual treatment. Putative secretion-limited or receptor-limited patterns require replication and cannot yet determine exercise mode, diet, or drug choice. Weight loss and training should be tested separately because reduced energy surplus does not necessarily restore muscle oxidative or neuromuscular adaptation.

Systemic therapeutic targeting is especially premature. gp130/LIFR signaling is pleiotropic; chronic exposure may trigger SOCS feedback, and the same ligand can have divergent muscle, neural, bone, adipose, immune, and cardiac effects [16,33,34,42,44,76,77,78,79]. Experimental work is, therefore, better directed toward source-resolved physiology and tissue-selective mechanisms than chronic systemic activation.

Broader organokine biology reinforces this caution. IL-6- and FGF21-related studies show that muscle–adipose–liver signaling can influence lipid metabolism and glucose transport, but directionality and source vary by context [80,81,82,83]. These adjacent studies support multi-tissue contextualization and analytical caution; they are not CNTF/CLCF1-specific evidence.

## 11. Outstanding Questions

The tiered roadmap prioritizes five questions: Which cells and tissues contribute CNTF, CLCF1, and CRLF1? Does aging alter extracellular release, receptor phosphorylation, or metabolic execution after accounting for adiposity and sex? Can physiological insulin or exercise pulses recruit the network at endogenous concentrations? Are ligand–receptor–metabolite kinetics reproducible within individuals? Finally, does tissue-specific disruption change the metabolic response, or is the network only a correlated marker? Negative and null results will be essential for refining or rejecting the framework.

## 12. Conclusions

What is known is limited: pharmacological CNTF can alter glucose and lipid handling, and one recent study links exercise-responsive CLCF1 to aging-related muscle and metabolic phenotypes. What is inferred is that extracellular ligand, receptor-proximal signaling, and metabolic execution may sometimes vary together across physiological states. What remains untested is whether this putative coupling is reproducible, muscle-derived, age-specific, necessary for adaptation, and measurable by human metabolomics or isotope tracing. Neurotrophic secretory flexibility should, therefore, be used as a falsifiable experimental framework, not as a validated biomarker or therapeutic target.

## Figures and Tables

**Table 1:** Selected core evidence inventory separating aging-specific evidence, exposure mode, and metabolic measurement. Category counts apply only to this purposive inventory; the table is not an exhaustive study-selection output.

Class/Study	Model, Age/Sex, Tissue	Exposure or Perturbation	Network and Metabolic Readout	Metabolomics or Flux	Boundary
Direct component-level: Kang et al. [16]	Human transcript reanalysis and six plasma cohorts; aged male mice; C2C12 myotubes; muscle, plasma, and bone.	Endogenous exercise response, recombinant CLCF1, genetic manipulation, and receptor knockdown; heterogeneous acute/chronic protocols.	CLCF1/CRLF1/receptors; glucose uptake, glycolytic flux, respiration, glucose tolerance, performance.	Targeted functional flux and respiration; no paired human muscle metabolomics or isotope tracing.	Only aging-focused bridge; human cohorts are exploratory and tissue source is unresolved.
Direct component-level: Watt et al. [13]	Obesity-related rodent and skeletal-muscle models; not aging-specific.	Recombinant or pharmacological CNTF; short-term experimental exposure.	AMPK activation, insulin sensitivity, glucose handling.	Targeted biochemical and physiological endpoints; no global metabolomics.	Pharmacological rescue does not establish endogenous secretion or physiological dose.
Direct component-level: Steinberg et al. [14]	Lean/obese muscle glucose-uptake models; not aging-specific.	Acute recombinant CNTF stimulation.	PI3K-dependent glucose uptake; obesity-associated response impairment.	Glucose uptake rather than downstream carbon-fate profiling.	Supports ligand resistance in obesity, not aging-muscle coupling.
Direct component-level: Watt et al. [15]	Acute lipid-challenge rodent/peripheral-tissue models; not aging-specific.	Recombinant CNTF during pharmacological lipid exposure.	Ceramide, JNK phosphorylation, and insulin action.	Targeted ceramide measurement; no paired secretomics or broad lipidomics.	Defines a lipid bottleneck under acute exposure, not chronic aging.
Partial mechanistic: Sato [30]	Cultured skeletal muscle cells; no age or sex context.	gp130 protein/*IL6ST* gene suppression during insulin stimulation.	Insulin signaling and glucose uptake.	No metabolomics or ligand kinetics.	Receptor-component evidence; ligand specificity is unresolved.
Partial mechanistic: Danowska et al. [31]	Human skeletal muscle in obesity/insulin-sensitivity phenotypes; age/sex reported in source.	Cross-sectional endogenous gene-expression study; no challenge.	*IL6ST* expression and metabolic phenotype.	No stimulated signaling, protein, or metabolomics.	Association in obesity cannot be treated as aging evidence.
Partial mechanistic: Fix et al. [32]	Mouse skeletal muscle under basal and exercise-trained conditions; not an aging study.	Muscle gp130-protein manipulation with training exposure.	Mitochondrial quality-control and adaptation endpoints.	Functional mitochondrial assays; not CNTF/ CLCF1-specific metabolomics.	Supports the shared receptor platform only.
Contextual boundary: Ding and Yuan et al. [33,34]	Brown adipocytes and mouse systemic-metabolism models; outside skeletal muscle.	CLCF1 exposure or pathway manipulation; study-specific doses and durations.	Thermogenesis, mitochondrial biogenesis, and energy expenditure.	Metabolic phenotyping outside muscle.	Opposing tissue effects argue against unqualified systemic translation.

**Table 2:** Operational criteria for testing neurotrophic secretory flexibility.

Layer	Minimum Measurement	Dynamic Metric	Critical Control	Observation That Weakens or Falsifies the Model
Production and source	CNTF/CLCF1/CRLF1 transcript and intracellular protein plus cell-type or spatial localization.	Basal value, fold change, peak, and recovery.	Fiber, stromal, endothelial, immune, neuronal, and non-muscle sources.	Plasma change without compatible local expression or source evidence.
Extracellular availability	Interstitial or arterial–venous ligand; free versus complexed CLCF1; soluble CNTFRα.	Peak, response area under the curve, time to peak, recovery, and within-person coefficient of variation.	Matrix effects, assay specificity, hemolysis, creatine kinase, myoglobin, and exercise injury.	Signal explained by leakage, assay cross-reactivity, or non-muscle release.
Receptor-proximal response	Paired phospho-STAT3, phospho-AKT, phospho-AMPK, or phospho-ERK with receptor protein and SOCS feedback.	Stimulus-to-basal response and temporal order relative to ligand.	Total protein, receptor shedding, shared gp130 ligands, dose, and prior exposure.	Ligand response repeatedly occurs without receptor-proximal activation.
Metabolic execution	At least one quantitative output: glucose uptake/traced fate, glycogen, lipid species/flux, respiration, or redox state.	Absolute and relative change, pathway score, or isotope-derived flux.	Workload, insulin, diet, adiposity, age, sex, circadian timing, and last exercise bout.	Receptor activation repeatedly occurs without a prespecified metabolic response.
Integrated construct	All three dynamic layers measured in the same unit and challenge, ideally on repeat visits.	Cross-lagged association, coupling coefficient with uncertainty, and test–retest reliability.	Prespecified direction, multiplicity control, and external replication.	Metabolic adaptation is preserved after tissue-specific disruption, or proposed ordering is not reproducible.

**Table 3:** Evidence tiers and analytical implications for metabolomic testing of the CNTF/CLCF1 network.

Evidence Tier	Currently Measured	Appropriate Analytical Extension	Permitted Inference
Direct component-level, network-specific	CNTF: glucose uptake, AMPK/PI3K signaling, ceramide, JNK, insulin action. CLCF1: glucose uptake, glycolytic flux, respiration, and glucose tolerance [13,14,15,16].	Targeted metabolite panels and isotope-resolved glucose/lipid flux synchronized with ligand and phosphorylation kinetics.	Component-level metabolic effects; not a validated human coupling system.
Partial mechanistic	gp130-protein or *IL6ST*-gene perturbation, receptor phosphorylation, mitochondrial quality control, and related gp130-ligand effects [30,31,32,43,45].	Phosphoproteomics plus targeted metabolomics in the same sample and stimulus.	Plausible receptor or signaling step; not CNTF/CLCF1 specificity.
Contextual aging/exercise	Frailty/sarcopenia muscle metabolomics, exercise plasma metabolomics, Lac-Phe, eicosanoids, recovery signals, and lifestyle-associated blood metabolic pathways in cognitive aging [11,46,47,55,56,57,58,59,60,61].	Paired muscle/plasma untargeted profiling with source-sensitive sampling and prespecified time courses.	Defines candidate modules and timing only; circulating changes do not prove muscle origin.
Prospective candidates	Acylcarnitines, ceramide and DAG species, TCA intermediates, NAD^+^ metabolites, glutathione/redox markers, BCAA catabolites, and mitochondrial lipids [6,8,10,12,51,52,53,54].	Targeted quantitative assays, lipid species resolution, redox-sensitive handling, and stable isotope tracing.	Biological plausibility and study-design priority; no direct CNTF/CLCF1 association should be claimed.

**Table 4:** Tiered validation roadmap integrating physiological state, sampling, and falsifiable comparisons.

Tier and State	Samples and Timing	Core Measures	Decisive Comparison or Falsifier
Tier 1: low-burden repeated response	Standardized fasting baseline plus multiple plasma samples during and after one defined exercise or meal/glucose challenge; repeat visit.	Free/complexed ligand where possible, creatine kinase/myoglobin, glucose/insulin, targeted metabolites, workload, and test–retest reliability.	Establishes kinetics and reproducibility; cannot prove muscle source or receptor competence.
Tier 2: paired tissue mechanism	Plasma plus muscle biopsy before and after ex vivo insulin or a standardized exercise bout; optional recovery biopsy.	Ligand/CRLF1 protein, receptor abundance, phospho-STAT3/AKT/AMPK/ERK, SOCS3, targeted or untargeted tissue metabolomics, respiration.	Tests temporal alignment. Ligand change without phosphorylation or metabolic output weakens integrated coupling.
Tier 3: source and flux attribution	Arterial–venous balance or microdialysis plus plasma and biopsy; stable isotope glucose/fatty-acid tracers across clamp or exercise.	Local release, ^13^C-carbon fate, lipid flux, acylcarnitines, ceramide/DAG species, mitochondrial/redox endpoints.	Distinguishes active muscle release and pathway flux from systemic concentration changes; invasive and best reserved for focused hypotheses.
Longitudinal intervention	Diet-only, aerobic, resistance, interval, or combined training with standardized last bout and matched age/adiposity strata.	Repeat the selected tier before and after intervention; muscle quality, insulin sensitivity, and adverse systemic effects.	Tests whether changes in putative coupling precede or mediate adaptation; preservation after tissue-specific disruption argues against necessity.

## Data Availability

No new data were created or analyzed in this review. Data sharing is not applicable to this article.

## Supplementary Materials

The following supporting information can be downloaded at: https://www.mdpi.com/article/10.3390/metabo16080562/s1, Table S1: Cohort-level summary of the exploratory human CLCF1 evidence reported by Kang et al. (2025) [16].

## Abbreviations

The following abbreviations are used in this manuscript:

ACC	Acetyl-CoA carboxylase
AICAR	5-aminoimidazole-4-carboxamide ribonucleotide
AKT	Protein kinase B
AMPK	AMP-activated protein kinase
AS160	Akt substrate of 160 kDa
BCAA	Branched-chain amino acid
BDNF	Brain-derived neurotrophic factor
CK	Creatine kinase
CLCF1	Cardiotrophin-like cytokine factor 1
CNTF	Ciliary neurotrophic factor
CNTFRα	Ciliary neurotrophic factor receptor alpha
CRLF1	Cytokine receptor-like factor 1
CV	Coefficient of variation
DAG	Diacylglycerol
ERK	Extracellular signal-regulated kinase
FFA	Free fatty acid
FGF21	Fibroblast growth factor 21
GLUT4	Glucose transporter type 4
gp130	Glycoprotein 130 protein encoded by *IL6ST*
*IL6ST*	Interleukin 6 signal transducer gene
IRS1	Insulin receptor substrate 1
JAK	Janus kinase
JNK	c-Jun N-terminal kinase
Lac-Phe	N-lactoyl-phenylalanine
LIFR	Leukemia inhibitory factor receptor
MOFA	Multi-omics factor analysis
NAD^+^	Nicotinamide adenine dinucleotide
OGTT	Oral glucose tolerance test
OXPHOS	Oxidative phosphorylation
PI3K	Phosphoinositide 3-kinase
PLS	Partial least squares
ROS	Reactive oxygen species
SOCS	Suppressor of cytokine signaling
SOCS3	Suppressor of cytokine signaling 3
STAT	Signal transducer and activator of transcription
TCA	Tricarboxylic acid

## Appendix A. Database Search Strategy

The search was designed for a targeted narrative review rather than a formal systematic review. All four databases were last searched on 30 June 2026. The retained PubMed export contained 695 records; exact final executed strings, record-level exports, and complete search histories were not retained for every database, so cross-database totals and duplicate counts cannot be reconstructed. No language filter was applied at retrieval. Detailed synthesis was restricted to peer-reviewed full articles in English or with sufficient accessible English-language text; conference abstracts, dissertations, preprints, inaccessible full texts, and non-English records without adequate English information were not used as core evidence. Screening was not performed as independent blinded duplicate review, no formal risk-of-bias instrument was applied, and uncertain relevance was discussed within the author team. Manual reference chasing of key articles and reviews supplemented database retrieval and contributed to the final evidence inventory. Table A1 documents representative platform queries reconstructed from the retained materials; they are not claimed to be verbatim final executed strings or a prospectively registered search audit trail.

**Table A1:** Representative database queries used to identify literature on CNTF/CLCF1 signaling, aging skeletal muscle, physiological challenges, and metabolomic outcomes. These strings are not a verbatim reproducible search audit trail.

Source	Coverage	Representative Query	Use in Synthesis
PubMed/MEDLINE	Biomedical studies from database inception to 30 June 2026	(CNTF OR “ciliary neurotrophic factor” OR CLCF1 OR “cardiotrophin-like cytokine factor 1” OR CRLF1 OR CNTFR OR LIFR OR gp130 OR IL6ST) AND (“skeletal muscle” OR myotube OR myocyte OR “muscle fiber” OR sarcopenia OR frailty OR aging OR ageing) AND (metabolomics OR lipidomics OR “glucose uptake” OR “glucose disposal” OR glycolysis OR ceramide OR acylcarnitine OR mitochondria OR redox OR “stable isotope” OR fluxomics OR exercise OR insulin OR “weight loss”)	Core biomedical retrieval, mechanistic studies, human exercise and aging-muscle studies.
Embase	Biomedical and pharmacological literature from database inception to 30 June 2026	(`ciliary neurotrophic factor’/exp OR CNTF OR CLCF1 OR `cardiotrophin like cytokine factor 1’ OR CRLF1 OR CNTFR OR LIFR OR gp130 OR IL6ST) AND (`skeletal muscle’/exp OR myotube OR sarcopenia OR frailty OR ageing OR aging) AND (`metabolomics’/exp OR lipidomics OR `glucose uptake’ OR glycolysis OR ceramide OR acylcarnitine OR mitochondria OR exercise OR insulin OR `stable isotope’)	Cross-checking pharmacological, receptor, and metabolic intervention records not always indexed identically in PubMed.
Web of Science Core Collection	Multidisciplinary citation database from database inception to 30 June 2026	TS = ((CNTF OR “ciliary neurotrophic factor” OR CLCF1 OR “cardiotrophin-like cytokine factor 1” OR CRLF1 OR CNTFR* OR LIFR OR gp130 OR IL6ST) AND (“skeletal muscle” OR myotube OR sarcopenia OR frailty OR aging OR ageing) AND (metabolomics OR lipidomics OR “glucose uptake” OR glycolysis OR ceramide OR acylcarnitine OR mitochondria OR redox OR exercise OR insulin OR fluxomics))	Citation expansion, identification of highly cited mechanistic work and recent multi-omics studies.
Scopus	Multidisciplinary abstract and citation database from database inception to 30 June 2026	TITLE-ABS-KEY((CNTF OR “ciliary neurotrophic factor” OR CLCF1 OR “cardiotrophin-like cytokine factor 1” OR CRLF1 OR CNTFR OR LIFR OR gp130 OR IL6ST) AND (“skeletal muscle” OR myotube OR sarcopenia OR frailty OR aging OR ageing) AND (metabolomics OR lipidomics OR “glucose uptake” OR glycolysis OR ceramide OR acylcarnitine OR mitochondria OR exercise OR insulin OR fluxomics))	Capture of recent exercise, exerkine, metabolomics, and translational physiology studies across journals.
Crossref and manual reference chasing	DOI and metadata checks; reference lists of key articles and reviews	DOI lookup was used for bibliographic verification where possible. Reference lists of key CNTF, CLCF1, gp130, myokine, exercise-metabolomics, and sarcopenia reviews were screened manually.	Verification of citation metadata and recovery of older canonical receptor or pharmacological studies.

## References

[1] Severinsen M.C.K., Pedersen B.K. (2020). Muscle-Organ Crosstalk: The Emerging Roles of Myokines. Endocr. Rev..

[2] Pedersen B.K., Febbraio M.A. (2008). Muscle as an endocrine organ: Focus on muscle-derived interleukin-6. Physiol. Rev..

[3] Shero J.A., Lindholm M.E., Sandri M., Stanford K.I. (2025). Skeletal Muscle as a Mediator of Interorgan Crosstalk During Exercise: Implications for Aging and Obesity. Circ. Res..

[4] Aslam M.A., Lee J., Bowen T.S., Huh J.Y. (2026). Exercise-induced myokines in metabolic regulation: Mechanisms, mimetics, and translational potential. Arch. Pharm. Res..

[5] Yi J., Chen J., Yao X., Zhao Z., Niu X., Li X., Sun J., Ji Y., Shang T., Gong L. (2025). Myokine-mediated muscle-organ interactions: Molecular mechanisms and clinical significance. Biochem. Pharmacol..

[6] Boardman N.T., Trani G., Scalabrin M., Romanello V., Wust R.C.I. (2023). Intracellular to interorgan mitochondrial communication in striated muscle in health and disease. Endocr. Rev..

[7] Jia D., Tian Z., Wang R. (2023). Exercise mitigates age-related metabolic diseases by improving mitochondrial dysfunction. Ageing Res. Rev..

[8] Li J., Wang Z., Li C., Song Y., Wang Y., Bo H., Zhang Y. (2022). Impact of exercise and aging on mitochondrial homeostasis in skeletal muscle: Roles of ROS and epigenetics. Cells.

[9] Romanello V. (2020). The interplay between mitochondrial morphology and myomitokines in aging sarcopenia. Int. J. Mol. Sci..

[10] Dantas W.S., Zunica E.R.M., Heintz E.C., Vandanmagsar B., Floyd Z.E., Yu Y., Fujioka H., Hoppel C.L., Belmont K.P., Axelrod C.L. (2022). Mitochondrial uncoupling attenuates sarcopenic obesity by enhancing skeletal muscle mitophagy and quality control. J. Cachexia Sarcopenia Muscle.

[11] Setoyama D., Han D., Tian J., Lee H.Y., Shin H.S., Nga H.T., Nguyen T.L., Moon J.S., Jang H.J., Kim E. (2025). Comparative analysis of primary sarcopenia and end-stage renal disease-related muscle wasting using multi-omics approaches. J. Cachexia Sarcopenia Muscle.

[12] Mengeste A.M., Rustan A.C., Lund J. (2021). Skeletal muscle energy metabolism in obesity. Obesity.

[13] Watt M.J., Dzamko N., Thomas W.G., Rose-John S., Ernst M., Carling D., Kemp B.E., Febbraio M.A., Steinberg G.R. (2006). CNTF reverses obesity-induced insulin resistance by activating skeletal muscle AMPK. Nat. Med..

[14] Steinberg G.R., Watt M.J., Ernst M., Birnbaum M.J., Kemp B.E., Jorgensen S.B. (2009). Ciliary neurotrophic factor stimulates muscle glucose uptake by a PI3-kinase-dependent pathway that is impaired with obesity. Diabetes.

[15] Watt M.J., Hevener A., Lancaster G.I., Febbraio M.A. (2006). Ciliary neurotrophic factor prevents acute lipid-induced insulin resistance by attenuating ceramide accumulation and phosphorylation of c-Jun N-terminal kinase in peripheral tissues. Endocrinology.

[16] Kang J.S., Kim J.H., Kim M.J., Min B., Lee S.M., Go G.Y., Kim J.W., Kim S., Kwak J.Y., Chun S.W. (2025). Exercise-induced CLCF1 attenuates age-related muscle and bone decline in mice. Nat. Commun..

[17] Forger N.G., Prevette D., deLapeyriere O., de Bovis B., Wang S., Bartlett P., Oppenheim R.W. (2003). Cardiotrophin-like cytokine/cytokine-like factor 1 is an essential trophic factor for lumbar and facial motoneurons in vivo. J. Neurosci..

[18] Derouet D., Rousseau F., Alfonsi F., Froger J., Hermann J., Barbier F., Perret D., Diveu C., Guillet C., Preisser L. (2004). Neuropoietin, a new IL-6-related cytokine signaling through the ciliary neurotrophic factor receptor. Proc. Natl. Acad. Sci. USA.

[19] Chung H.S., Choi K.M. (2026). Exercise, exerkines, and sarcopenia. Diabetes Metab. J..

[20] Cui X., Liu H., Liu Y., Yu Z., Wang D., Wei W., Wang S. (2025). The role and mechanisms of myokines in sarcopenia: New intervention strategies for the challenges of aging. Front. Med..

[21] Luo X., Wang J., Ju Q., Li T., Bi X. (2025). Molecular mechanisms and potential interventions during aging-associated sarcopenia. Mech. Ageing Dev..

[22] Nguyen T.T., Dao T., Nguyen H.T., Park J.H., Jeong S.J., Kim S., Jo Y., Thieu N.T.H., Zhao J., Ding F. (2026). Sarcopenia and muscle aging: Updated insights into molecular mechanisms and translational therapeutics. Endocrinol. Metab..

[23] Dao T., Green A.E., Kim Y.A., Bae S.J., Ha K.T., Gariani K., Lee M.R., Menzies K.J., Ryu D. (2020). Sarcopenia and muscle aging: A brief overview. Endocrinol. Metab..

[24] Wang J., Jia D., Zhang Z., Wang D. (2025). Exerkines and sarcopenia: Unveiling the mechanism behind exercise-induced mitochondrial homeostasis. Metabolites.

[25] Barros D., Marques E.A., Magalhaes J., Carvalho J. (2022). Energy metabolism and frailty: The potential role of exercise-induced myokines—A narrative review. Ageing Res. Rev..

[26] Alizadeh Pahlavani H. (2022). Exercise therapy for people with sarcopenic obesity: Myokines and adipokines as effective actors. Front. Endocrinol..

[27] Shao H., Zhang H., Jia D. (2024). The role of exerkines in obesity-induced disruption of mitochondrial homeostasis in thermogenic fat. Metabolites.

[28] Watkins B.A., Smith B.J., Volpe S.L., Shen C.L. (2024). Exerkines, nutrition, and systemic metabolism. Nutrients.

[29] Saponaro F., Bertolini A., Baragatti R., Galfo L., Chiellini G., Saba A., D’Urso G. (2024). Myokines and microbiota: New perspectives in the endocrine muscle-gut axis. Nutrients.

[30] Sato K. (2024). Suppression of gp130 attenuated insulin-mediated signaling and glucose uptake in skeletal muscle cells. Sci. Rep..

[31] Danowska M., Nikolajuk A., Straczkowski M. (2026). Skeletal muscle expression of the genes associated with glycoprotein 130 signaling in relation to obesity and insulin sensitivity. J. Endocrinol. Investig..

[32] Fix D.K., Hardee J.P., Gao S., VanderVeen B.N., Velazquez K.T., Carson J.A. (2018). Role of gp130 in basal and exercise-trained skeletal muscle mitochondrial quality control. J. Appl. Physiol..

[33] Ding M., Xu H.Y., Zhou W.Y., Xia Y.F., Li B.Y., Shi Y.J., Dou X., Yang Q.Q., Qian S.W., Tang Y. (2023). CLCF1 signaling restrains thermogenesis and disrupts metabolic homeostasis by inhibiting mitochondrial biogenesis in brown adipocytes. Proc. Natl. Acad. Sci. USA.

[34] Yuan Y., Li K., Ye X., Wen S., Zhang Y., Teng F., Zhou X., Deng Y., Yang X., Wang W. (2024). CLCF1 inhibits energy expenditure via suppressing brown fat thermogenesis. Proc. Natl. Acad. Sci. USA.

[35] Sleeman M.W., Anderson K.D., Lambert P.D., Yancopoulos G.D., Wiegand S.J. (2000). The ciliary neurotrophic factor and its receptor, CNTFR alpha. Pharm. Acta Helv..

[36] Vergara C., Ramirez B. (2004). CNTF, a pleiotropic cytokine: Emphasis on its myotrophic role. Brain Res. Brain Res. Rev..

[37] Guillet C., Auguste P., Mayo W., Kreher P., Gascan H. (1999). Ciliary neurotrophic factor is a regulator of muscular strength in aging. J. Neurosci..

[38] Weis J., Lie D.C., Ragoss U., Zuchner S.L., Schroder J.M., Karpati G., Farruggella T., Stahl N., Yancopoulos G.D., DiStefano P.S. (1998). Increased expression of CNTF receptor alpha in denervated human skeletal muscle. J. Neuropathol. Exp. Neurol..

[39] Kraakman M.J., Allen T.L., Whitham M., Iliades P., Kammoun H.L., Estevez E., Lancaster G.I., Febbraio M.A. (2013). Targeting gp130 to prevent inflammation and promote insulin action. Diabetes Obes. Metab..

[40] Febbraio M.A. (2007). gp130 receptor ligands as potential therapeutic targets for obesity. J. Clin. Investig..

[41] Steinberg G.R., Jorgensen S.B. (2007). The AMP-activated protein kinase: Role in regulation of skeletal muscle metabolism and insulin sensitivity. Mini Rev. Med. Chem..

[42] Cron L., Allen T., Febbraio M.A. (2016). The role of gp130 receptor cytokines in the regulation of metabolic homeostasis. J. Exp. Biol..

[43] Brandt N., O’Neill H.M., Kleinert M., Schjerling P., Vernet E., Steinberg G.R., Richter E.A., Jorgensen S.B. (2015). Leukemia inhibitory factor increases glucose uptake in mouse skeletal muscle. Am. J. Physiol. Endocrinol. Metab..

[44] Findeisen M., Allen T.L., Henstridge D.C., Kammoun H., Brandon A.E., Baggio L.L., Watt K.I., Pal M., Cron L., Estevez E. (2019). Treatment of type 2 diabetes with the designer cytokine IC7Fc. Nature.

[45] VanderVeen B.N., Fix D.K., Montalvo R.N., Counts B.R., Smuder A.J., Murphy E.A., Koh H.J., Carson J.A. (2019). The regulation of skeletal muscle fatigability and mitochondrial function by chronically elevated interleukin-6. Exp. Physiol..

[46] Morville T., Sahl R.E., Moritz T., Helge J.W., Clemmensen C. (2020). Plasma metabolome profiling of resistance exercise and endurance exercise in humans. Cell Rep..

[47] Weber D., Ferrario P.G., Bub A. (2025). Exercise intensity determines circulating levels of Lac-Phe and other exerkines: A randomized crossover trial. Metabolomics.

[48] Colleluori G., Viola V., Bathina S., Armamento-Villareal R., Qualls C., Giordano A., Villareal D.T. (2025). Effect of aerobic or resistance exercise, or both on insulin secretion, ciliary neurotrophic factor, and insulin-like growth factor-1 in dieting older adults with obesity. Clin. Nutr..

[49] Gonzalez-Franquesa A., Peijs L., Cervone D.T., Kocana C., Zierath J.R., Deshmukh A.S. (2021). Insulin and 5-aminoimidazole-4-carboxamide ribonucleotide (AICAR) differentially regulate the skeletal muscle cell secretome. Proteomes.

[50] Tok O., Kisioglu S.V., Ersoz H.O., Kahveci B., Goktas Z. (2021). Effects of increased physical activity and/or weight loss diet on serum myokine and adipokine levels in overweight adults with impaired glucose metabolism. J. Diabetes Complicat..

[51] Jang S.Y., Choi K.M. (2025). Impact of adipose tissue and lipids on skeletal muscle in sarcopenia. J. Cachexia Sarcopenia Muscle.

[52] Pollak N., Janezic E.G., Sink Z., Ugwoke C.K. (2025). Crosstalk between skeletal muscle and proximal connective tissues in lipid dysregulation in obesity and type 2 diabetes. Metabolites.

[53] Sutton E., Pekovic-Vaughan V. (2025). Time to reset: The interplay between circadian rhythms and redox homeostasis in skeletal muscle ageing and systemic health. Antioxidants.

[54] Caporossi D., Jackson M.J., Henriquez-Olguin C. (2026). Intracellular and extracellular redox signals during exercise and aging. Free Radic. Biol. Med..

[55] Fazelzadeh P., Hangelbroek R.W.J., Tieland M., de Groot L.C.P.G.M., Verdijk L.B., van Loon L.J.C., Smilde A.K., Alves R.D.A.M., Vervoort J., Muller M. (2016). The muscle metabolome differs between healthy and frail older adults. J. Proteome Res..

[56] Li K., Schon M., Naviaux J.C., Monk J.M., Alchus-Laiferova N., Wang L., Straka I., Matejicka P., Valkovic P., Ukropec J. (2022). Cerebrospinal fluid and plasma metabolomics of acute endurance exercise. FASEB J..

[57] Ambatipudi M., Roshandelpoor A., Guseh J.S., Kosyakovsky L.B., Alotaibi M., Cheng S., Jain M., Lewis G.D., Ho J.E. (2025). The role of bioactive lipids and eicosanoid metabolites in acute exercise in adults: Insights into human cardiorespiratory fitness. Physiol. Rep..

[58] Barlow J.P., Karstoft K., Vigelso A., Gram M., Helge J.W., Dela F., Pappan K., O’Neil D., Dunn W., Solomon T.P.J. (2020). Beta-aminoisobutyric acid is released by contracting human skeletal muscle and lowers insulin release from INS-1 832/3 cells by mediating mitochondrial energy metabolism. Metabol. Open.

[59] Chan W.S., Ng C.F., Pang B.P.S., Hang M., Tse M.C.L., Iu E.C.Y., Ooi X.C., Yang X., Kim J.K., Lee C.W. (2024). Exercise-induced BDNF promotes PPAR*δ*-dependent reprogramming of lipid metabolism in skeletal muscle during exercise recovery. Sci. Signal..

[60] Nam J.S., Park S.J., Ahn C.W., Cho E.S., Kim H.J., Kim Y. (2024). Follistatin-like 1 is a myokine regulating lipid mobilization during endurance exercise and recovery. Obesity.

[61] Chen Y., Gui H., Ma K., Zhang Z., Zhao T., Wang M. (2026). Lifestyle-associated blood metabolic pathways and functional performance in cognitive aging. Sci. Rep..

[62] Mastrototaro L., Roden M. (2021). Insulin resistance and insulin sensitizing agents. Metabolism.

[63] Feraco A., Gorini S., Armani A., Camajani E., Rizzo M., Caprio M. (2021). Exploring the role of skeletal muscle in insulin resistance: Lessons from cultured cells to animal models. Int. J. Mol. Sci..

[64] Balakrishnan R., Thurmond D.C. (2022). Mechanisms by which skeletal muscle myokines ameliorate insulin resistance. Int. J. Mol. Sci..

[65] Ahsan M., Garneau L., Aguer C. (2022). The bidirectional relationship between AMPK pathway activation and myokine secretion in skeletal muscle: How it affects energy metabolism. Front. Physiol..

[66] Huang Y., Wang C., Cui H., Sun G., Qi X., Yao X. (2025). Mitochondrial dysfunction in age-related sarcopenia: Mechanistic insights, diagnostic advances, and therapeutic prospects. Front. Cell Dev. Biol..

[67] Deng Q., Huang J., Wang C., Liang J. (2026). Exercise-induced exerkines modulate autophagy: Implications for interorgan crosstalk in the hallmarks of ageing. Int. J. Mol. Sci..

[68] Yano N., Zhang L., Wei D., Dubielecka P.M., Wei L., Zhuang S., Zhu P., Qin G., Liu P.Y., Chin Y.E. (2020). Irisin counteracts high glucose and fatty acid-induced cytotoxicity by preserving the AMPK-insulin receptor signaling axis in C2C12 myoblasts. Am. J. Physiol. Endocrinol. Metab..

[69] Lee J.O., Byun W.S., Kang M.J., Han J.A., Moon J., Shin M.J., Lee H.J., Chung J.H., Lee J.S., Son C.G. (2020). The myokine meteorin-like improves glucose tolerance in both skeletal muscle cells and mice by targeting AMPK*α*2. FEBS J..

[70] Chen Y., Zhu J., Gui H., Liu M., Zhang Y., Wu Z., Liu C., Wang M. (2026). Linking gut microbiota, mitochondrial redox dysfunction, and ferroptosis in cardiometabolic diseases: A narrative review of mechanistic evidence and redox-targeted interventions. Antioxidants.

[71] Kruse R., Vienberg S.G., Vind B.F., Andersen B., Hojlund K. (2017). Effects of insulin and exercise training on FGF21, its receptors and target genes in obesity and type 2 diabetes. Diabetologia.

[72] Hojman P., Pedersen M., Nielsen A.R., Krogh-Madsen R., Yfanti C., Akerstrom T., Nielsen S., Pedersen B.K. (2009). Fibroblast growth factor-21 is induced in human skeletal muscles by hyperinsulinemia. Diabetes.

[73] Garneau L., Mulvihill E.E., Smith S.R., Sparks L.M., Aguer C. (2024). Myokine secretion following an aerobic exercise intervention in individuals with type 2 diabetes with or without exercise resistance. Int. J. Mol. Sci..

[74] Shakoor H., Kizhakkayil J., Statsenko Y., Platat C. (2025). Separate and combined effects of moderate-intensity exercise training and detraining with protocatechuic acid on myokines and insulin-signaling pathways in male Wistar rats. Metabolites.

[75] Chen Y., Gui H., Zhao T., Liu C., Zhang Y., Wang M., Yang R. (2026). Ultra-processed foods, MASLD, and cognitive aging: A processing-centered gut–liver–brain axis perspective. Nutrients.

[76] Gao S., Durstine J.L., Koh H.J., Carver W.E., Frizzell N., Carson J.A. (2017). Acute myotube protein synthesis regulation by IL-6-related cytokines. Am. J. Physiol. Cell Physiol..

[77] Reihmane D., Dela F. (2014). Interleukin-6: Possible biological roles during exercise. Eur. J. Sport Sci..

[78] Fisman E.Z., Tenenbaum A. (2010). The ubiquitous interleukin-6: A time for reappraisal. Cardiovasc. Diabetol..

[79] Galli C., Colleluori G., Perugini J., Scopini E., Severi I., Grandin G., Giordano A. (2026). The ciliary neurotrophic factor induces Stat3 phosphorylation in distinctive cytotypes of organs involved in body metabolism: An immunohistochemical study. J. Anat..

[80] Nara H., Watanabe R. (2021). Anti-inflammatory effect of muscle-derived interleukin-6 and its involvement in lipid metabolism. Int. J. Mol. Sci..

[81] Lin W., Song H., Shen J., Wang J., Yang Y., Cao J., Xue L., Zhao F., Xiao T., Lin R. (2023). Functional role of skeletal muscle-derived interleukin-6 and its effects on lipid metabolism. Front. Physiol..

[82] Rosales-Soto G., Diaz-Vegas A., Casas M., Contreras-Ferrat A., Jaimovich E. (2020). Fibroblast growth factor-21 potentiates glucose transport in skeletal muscle fibers. J. Mol. Endocrinol..

[83] Lorincz H., Somodi S., Ratku B., Harangi M., Paragh G. (2023). Crucial regulatory role of organokines in relation to metabolic changes in non-diabetic obesity. Metabolites.

