# Supplementary material for: A Stimulus-Resolved Framework for Investigating Putative Neurotrophic Secretome–Metabolome Coupling in Aging Skeletal Muscle: CNTF and CLCF1 as Non-Equivalent Nodes
Source: Metabolites. 2026 Aug 9;16(8):562. doi: 10.3390/metabo16080562 (PMC13515463; doi:10.3390/metabo16080562)
Supplement: Supplementary file 1 [file metabolites-16-00562-s001.zip › Supplementary_Material.pdf]

Supplementary Material

A Stimulus-Resolved Framework for Investigating Putative Neurotrophic Secretome–Metabolome Coupling in Aging Skeletal Muscle

Supplementary Table S1. Cohort-level summary of the exploratory human CLCF1 evidence reported by Kang et al. (2025). Values are reproduced from the published article and supplementary methods. Ages are reported as mean ± SD where a range was unavailable. EDTA, ethylenediaminetetraacetic acid; ELISA, enzyme-linked immunosorbent assay; HIIT, high-intensity interval training; HR_{max}, maximum heart rate; NR, not reported; 1-RM, one-repetition maximum.

Human cohort	Sample size, sex, and age	Exercise modality	Sampling time points	Biological material and assay	Information unavailable or interpretive boundary
1. Young adults, acute resistance exercise	$n = 10$; men; 25.33 ± 1.84 years (range NR)	Moderate-intensity resistance exercise, 50–69% 1-RM; approximately 1 h	Before exercise and 1 h after exercise; fasting ≥8 h	EDTA plasma; CLCF1 ELISA; samples assayed in duplicate	Exact age range and participant-level training history NR; plasma response does not establish muscle source
2. Young jiu-jitsu athletes, acute HIIT	n NR; sex NR; 28.25 ± 3.62 years (range NR)	Four rounds of jiu-jitsu-specific high-intensity interval resistance exercise designed to mimic match conditions	Before exercise and immediately after the fourth round	Plasma; CLCF1 ELISA; samples assayed in duplicate	Sample size, sex, exact age range, and fasting status NR; highly trained competitive cohort had elevated baseline CLCF1
3. Young adults, acute aerobic exercise	$n = 10$; men; 25.98 ± 1.74 years (range NR)	Treadmill exercise for 60 min at 40–59% HR_{max}	Before exercise and 1 h after exercise; fasting ≥8 h	EDTA plasma; CLCF1 ELISA; samples assayed in duplicate	Exact age range and detailed training history NR; no significant post-exercise increase was observed
4. Older adults, chronic resistance training	$n = 27$; women; 72.32 ± 0.95 years (range NR)	Resistance training at 30–49% 1-RM, 1 h/day, 5 days/week for 16 weeks	Blood collected the day after the final session following fasting ≥8 h; baseline timing not fully detailed in the main article	EDTA plasma; CLCF1 ELISA; samples assayed in duplicate	Exact age range and participant-level adherence NR; female-only cohort limits sex generalization
5. Older adults, chronic resistance-band training	$n = 14$; sex NR; 75.0 ± 3.46 years (range NR)	Elastic-band high-speed power training, two 60-min sessions/week for 12 weeks	Before and after the 12-week program; post-training blood collected the day after the final session following fasting ≥8 h	EDTA plasma; CLCF1 ELISA; samples assayed in duplicate	Sex and exact age range NR; samples originated from a previously published intervention cohort
6. Basal young-versus-older comparison	Pre-exercise samples from cohorts 1–5 plus 16 additional young and 10 additional older participants; sex NR; additional older group 73.36 ± 4.69 years; other ages/ranges NR	No acute exercise; basal cross-sectional comparison	Pre-exercise or non-exercise basal sampling; exact alignment across source cohorts NR	EDTA plasma; CLCF1 ELISA; samples assayed in duplicate	Total non-overlapping sample size, sex distribution, young-group age, exact age ranges, and harmonized sampling conditions NR

Source: Kang, J.S. et al. Exercise-induced CLCF1 attenuates age-related muscle and bone decline in mice. *Nature Communications* 2025, 16, 4743. doi:10.1038/s41467-025-59959-w.
